# Identification of S100A8 as a common diagnostic biomarkers and exploring potential pathogenesis for osteoarthritis and metabolic syndrome

**DOI:** 10.3389/fimmu.2023.1185275

**Published:** 2023-07-11

**Authors:** Xu Huang, Jiacheng Liu, Wei Huang

**Affiliations:** ^1^ Department of Critical Care Medicine, The First Affiliated Hospital of Chongqing Medical University, Chongqing, China; ^2^ Department of Orthopedics, Orthopedic Laboratory of Chongqing Medical University, The First Affiliated Hospital of Chongqing Medical University, Chongqing, China

**Keywords:** osteoarthritis, metabolic syndrome, S100A8, diagnostic biomarker, GEO

## Abstract

**Background:**

Osteoarthritis (OA) is the most frequent musculoskeletal disease and the major contributor to disability worldwide. Metabolic syndrome (MetS) has been recognized as being associated with the pathogenesis of osteoarthritis. However, the exact mechanisms and links between the two are not clear.

**Methods:**

We downloaded clinical information data and gene expression profiles for OA and MetS from the database of Gene Expression Omnibus (GEO), and immune related gene (IRG) from the database of Immunology Database and Analysis Portal (IMMPORT). After screening OA-DEG and MetS-DEG, we identified the common immune hub gene by screening the overlapping genes between OA-DEG, MetS-DEG and IRG. Then we conducted single-gene analysis of S100A8, assessed the correlation of S100A8 with immune cell infiltration, and verified the diagnostic value of S100A8 in OA and MetS database respectively.

**Results:**

323 OA-DEGs,101 MetS-DEGs and an immune-related hub gene, S100A8, were identified. In single gene analysis of S100A8 in OA samples, GSEA suggested that immune-related biological processes were more significantly enriched. The results of immune cell infiltration analysis showed that the enrichment fraction of M2 macrophages was significantly higher in the high S100A8-expressing group, and the level of S100A8 expression was positively correlated with M2 macrophage infiltration. The results of the dataset validation showed that S100A8 expression levels were significantly upregulated in the OA group and performed well in the diagnosis of OA. In single gene analysis of S100A8 in MetS samples, immune cell infiltration analysis showed that monocyte infiltration was higher in the S100A8 high expression samples and that there was a positive correlation between the two. Dataset validation showed that S100A8 is of high value for the diagnosis of MetS. In the validation of the dataset for the four metabolism-related diseases (obesity, diabetes, hypertension and hyperlipidaemia), S100A8 was expressed at higher levels in the disease group and also had a higher diagnostic value for the four metabolism-related diseases.

**Conclusion:**

S100A8 is a common hub gene and diagnostic biomarker for OA and MetS, and the immune regulation involved in S100A8 may play a central role in the pathogenesis of OA and MetS.

## Introduction

Osteoarthritis (OA) is the main reason for disability and the most frequent musculoskeletal disorder worldwide (1), and it affects a larger proportion of people over the age of 70 and increases the risk of co-morbidity and death (2). OA is characterized by synovitis, remodeling of the underlying bone and degeneration of articular cartilage (3, 4), which ultimately leads to joint function loss. OA leads to a high incidence of polyarticular involvement and typically affects the hands, feet, spine, hips and knees. The etiology of OA is related to several conditions including aging, metabolic factors, immune factors and mechanical factors (5, 6).

Metabolic syndrome (MetS) characterized by obesity, hypertension, hyperlipidemia and diabetes has been recognized as closely related to the pathogenesis of OA (7). The strong correlation between MetS and OA has also been confirmed in epidemiological studies (8). Obesity leads to OA in two ways, a weight-dependent way with increased mechanical loading of the joints and a weight-independent way which leads to systemic and local inflammation (9). Some studies have shown that hypertension increase the risk of OA development (10). In addition, two meta-analyses also confirmed a positive correlation between hyperlipidemia and OA (11, 12). Some researchers also found that subchondral bone loss was greater in patients with hypertension and diabetes compared to those without these conditions (13).

So, a growing amount of research suggested that there was a close relationship between OA and MetS, and that there may be common pathways and key genes in the pathogenesis of both. However, to date, the common pathogenesis of OA and MetS was unclear and few studies have been conducted to explore the molecular mechanisms of association between OA and MetS based on bioinformatics analysis. We explored common hub gene between OA and MetS and further performed single gene analysis and explored its relationship with immune infiltrating cells to understand the pathogenesis of OA and MetS. We then validated the common hub gene in multiple datasets to understand its diagnostic value for OA and MetS, helping to identify potential diagnostic biological markers and treatment candidates for both.

## Materials and methods

### Microarray data

Gene expression comprehensive database (GEO) is the most complete and largest public gene expression data resource worldwide, so we downloaded the dataset from GEO for analysis. The gene expression profiling dataset GSE114007 included 18 normal and 20 OA cartilage tissue of the human knee joint, and dataset GSE89408 included 28 normal and 22 OA human joint synovial biopsy. In GSE114007 and GSE89408, gene set enrichment analysis (GSEA) was applied to verify the correlation between OA and MetS. The datasets GSE98918, GSE82107, GSE117999 and GSE1919 as validation sets include 12 OA and 12 controls, 10 OA and 7 controls, 12 OA and 12 controls, 5 OA and 5 controls, respectively. The array-based gene expression profile dataset GSE23561 collected peripheral blood samples from 9 normal subjects and 6 Metabolic Syndrome. Other metabolic related disease datasets GSE161042, GSE15932, GSE76845, GSE1010 include 8 Obese Patients and 5 normal, 8 diabetes mellitus and 8 normal, 5 hypertension patients and 5 normal, 12 hyperlipemia patients and 12 normal, respectively. The details of the above datasets are shown in **Table 1**.

**Table 1:** Information of all datasets in the paper.

ID	GSE number	Platform	Samples	Disease	Group
1	GSE114007	GPL11154	20 osteoarthritis and 18 controls	Osteoarthritis	Discovery cohort
2	GSE89408	GPL11154	22 osteoarthritis and 28 controls	Osteoarthritis	Discovery cohort
3	GSE98918	GPL20855	12 osteoarthritis and 12 controls	Osteoarthritis	Validation cohort
4	GSE82107	GPL570	10 osteoarthritis and 7 controls	Osteoarthritis	Validation cohort
5	GSE117999	GPL20844	12 osteoarthritis and 12 controls	Osteoarthritis	Validation cohort
6	GSE1919	GPL9 91	5 osteoarthritis and 5 controls	Osteoarthritis	Validation cohort
7	GSE23561	GPL10775	6 metaboic syndrome and 9 controls	Metabolic syndrome	Discovery cohort
8	GSE161042	GPL18573	8 obese Patients and 5 controls	Metabolic related diseases	Validation cohort
9	GSE15932	GPL570	8 diabetesm ellitus and 8 controls	Metabolic related diseases	Validation cohort
10	GSE76845	GPL13825	5 hypertension patients and 5 controls	Metabolic related diseases	Validation cohort
11	GSE1010	GPL96	12 hyperlipem ia patients and 12 controls	Metabolic related diseases	Validation cohort

### Screening for differentially expressed genes

We screened the DEG between OA patients and control individuals in the two datasets GSE114007 and GSE89408, and the DEG between MetS patients and control individuals in the dataset GSE23561 through the “limma” package. We used the “ggplot2” software package to draw the volcano map of DEG to show the differential expression. The following criteria were statistically significant: P<0.05 and | log2FC |>1.

We performed subsequent Kyoto Encyclopedia of Genes and Genomes (KEGG) pathway and Gene Ontology (GO) enrichment analysis on the DEGs of each of the above three datasets using R software (4.2.1) and cluster analysis package.

The overlapping genes between the DEGs of GSE114007 and the DEGs of GSE89408 was identified as OA-DEGs, and the DEGs of GSE 23561 was identified as MetS-DEGs. The analysis of PPI, MCODE and the function enrichment of OA-DEGs were obtained by The Metascape database (http://metascape.org/).

### Screening of immune-related hub DEGs

Through the ImmPort database (https://www.immport.org/home), we obtained 2483 immune related genes (IRGs) totally. Then, S100A8 was identified by screening the overlapping genes among IRG, OA-DEG and MetS-DEG through Venn map, that is, immune related hub DEG (IRDEG).

### Building protein–protein interaction network

Totally 50 S100A8-binding proteins were acquired from the STRING network (https://string-db.org/) by setting the following main parameters: minimum required interaction score [“medium confidence (0.400)”] and active interaction sources (“Experiments, Text mining, Databases”). Visualization of the PPI network was then obtained by Cytoscape (version 3.7.2). We conducted the pathway enrichment analysis for GO and KEGG of 50 S100A8-binding proteins by a cluster Profiler package for statistical analysis and visualized them with the “ggplot2” package.

### Single-gene analysis for the S100A8 in OA database

#### Analysis of DEGs

On the basis of S100A8 expression median value, OA samples in datasets GSE114007 and GSE89408 were classified into S100A8 high expression group and S100A8 low expression group. DEGs between these two groups were analyzed by R package DESeq2 and volcano plots to show differential expression were plotted by the “ggplot2” package (Statistical significance was set at p-values less than 0.05 and |log2FC| greater than 1).

#### Gene set enrichment analysis

To identify biological signal pathways, GSEA was performed between high and low levels of S100A8 expression in OA samples of GSE114007 and GSE89408 datasets. The KEGG pathway was demonstrated to have significant enrichment results on the basis of net enrichment scores (NES), gene ratios and P values. |NES|>1 and FDR q<0.25 were considered significantly enriched. Statistical analysis were conducted using the R software (4.2.1) and the results were visualized with the “ggplot2” package.

#### Correlation between S100A8 and immune cell infiltrates

22 immune cells in OA samples are quantified by “CIBERSORT” software package, and the data with CIBERSORT value of p<0.05 were included in a more detailed analysis. Therefore, we obtained a matrix of immune cell fractions. Wilcoxon rank sum test was used to assess the difference in the level of immune infiltration between S100A8 high expression group and low expression group. Software R (4.2.1) package “boxplot” was utilized to show the difference in immune cell infiltration between S100A8 high and low expression group. Finally, Spearman correlation analysis was used to examine the association of S100A8 expression with immune cell infiltration, and the R(4.2.1) package “ggplot2” was used for visualization.

#### Validation of S100A8 expression and diagnostic value

The mRNA expression of S100A8 was verified in GSE114007, GSE89408, GSE98918, GSE82107, GSE117999, GSE1919. The comparison between the OA patients and controls was conducted with the T-test. Statistical significance was set at p-values less than 0.05.

We determined the sensitivity and specificity of S100A8 by performing Receiver Operating Characteristic (ROC) curve analysis with the R (4.2.1) pROC package, visualized using the R package “ggplot2”.

### Single-gene analysis for the S100A8 in MetS database

#### Diagnostic value and immune infiltration analysis of S100A8

The ROC curve was applied to evaluate the diagnosis value of S100A8 in MetS patients, which was performed using R (4.2.1) pROC package and visualized using R package “ggplot2”.

On the basis of median value of S100A8 level, MetS samples in GSE23561 were classified into S100A8 high group and S100A8 low group. The infiltration degrees of 22 immune cell were compared between S100A8 high level group and S100A8 low level group. The visualization of the differences was conducted using R software package. We explored the association of S100A8 expression with immune cell infiltration using Spearman’s correlation analysis, and used R package “ggplot2” for visualization.

#### Validation of S100A8 expression and diagnostic value in metabolic related diseases

The mRNA expression of S100A8 was verified in 4 gene expression profiles of patients with Metabolic related diseases (GSE161042, GSE15932, GSE76845, GSE1010). The GSE161042 dataset contained 8 Obese Patient and 5 controls, the GSE15932 dataset contained 8 diabetes mellitus and 8 controls, the GSE76845 dataset contained 5 hypertension and 5 controls, the GSE1010 dataset contained 12 hyperlipemia patients and 12 controls. Comparison of S100A8 expression level between patients and controls was performed by t-test. P-value < 0.05 was considered significant. Sensitivity and specificity of S100A8 in the four metabolic related diseases mentioned above were assessed by ROC curves.

#### Associations between S100A8 expression and body mass index

We analyzed the correlation between S100A8 expression level and BMI in the dataset GSE161042, GSE117999 and GSE98918. We used R (4.2.1) for the statistics analysis, visualized using the R “ggplot2” package.

## Result

### Identification of DEGs and analysis of GO and KEGG pathway enrichment

**Figure 1:**
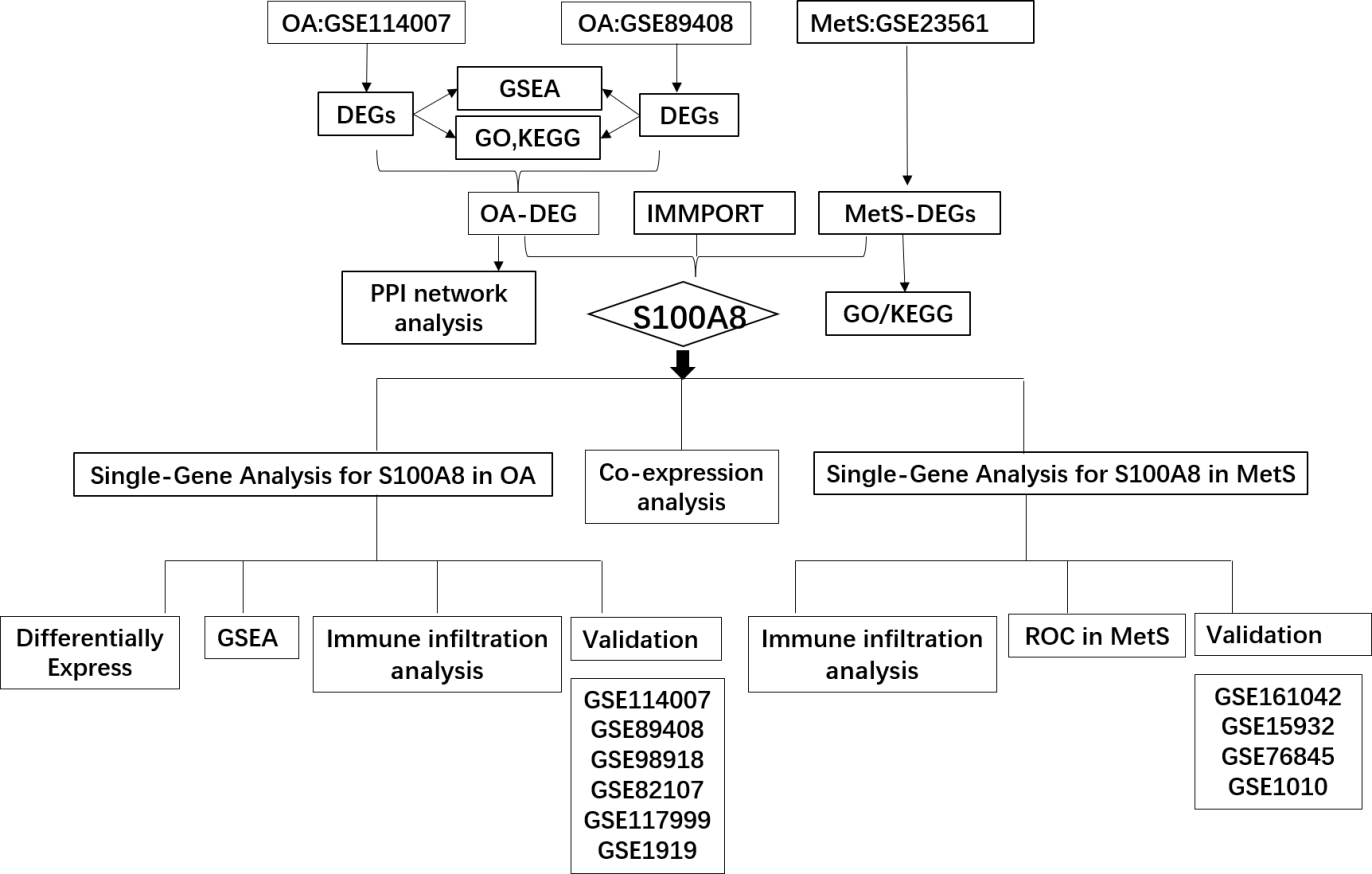
The flow chart of this study.

**Figure 2:**
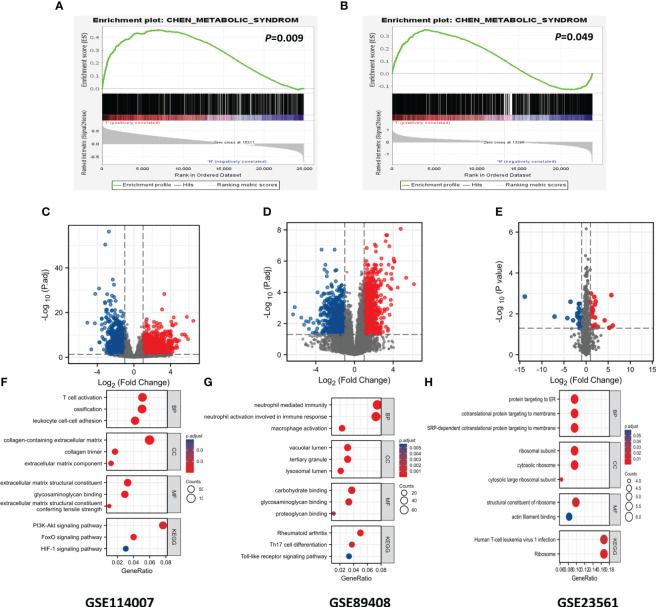
GSEA analysis, DEGs identification and GO and KEGG analysis. **(A)** Using GSEA analysis to verify the correlation between OA and MS in GSE89408. **(B)** Using GSEA analysis to verify the correlation between OA and MS in GSE114007. **(C)** Identification of DEGs in GSE114007. **(D)** Identification of DEGs in GSE89408. **(E)** Identification of DEGs in GSE23561. **(F)** GO and KEGG terms of DGEs in GSE114007. **(G)** GO and KEGG terms of DGEs in GSE89408. **(H)** GO and KEGG terms of DGEs in GSE23561.

The diagram of the research design flow is presented in **Figure 1**. We initially identified 2247 DEGs between OA patients and control individuals in GSE114007, and 1475 DEGs in GSE89408. To understand the relationship between OA and MetS, based on the KEGG gene set in MetSigDB database, we conducted GSEA analysis and found that OA gene set was significantly enriched in MetS (P=0.009 in GSE89408, p=0.049 in GSE114007) (**Figures 2A, B**). We further obtained 101 MetS-DEGs between MetS patients and control individuals in GSE23561. The volcano plot of DEGs were shown in **Figures 2C–E**. The DEGs in the above three datasets were utilized for GO and KEGG analysis. The top 3 enriched GO terms were listed in **Figures 2F–H**. In GSE114007 and GSE89408, the most significant BP terms were about Immune cell. We also detected that DEGs were significantly enriched in the Immune and inflammatory mediator pathway in the 3 datasets.

### Screening and analysis of OA-DEGs

**Figure 3:**
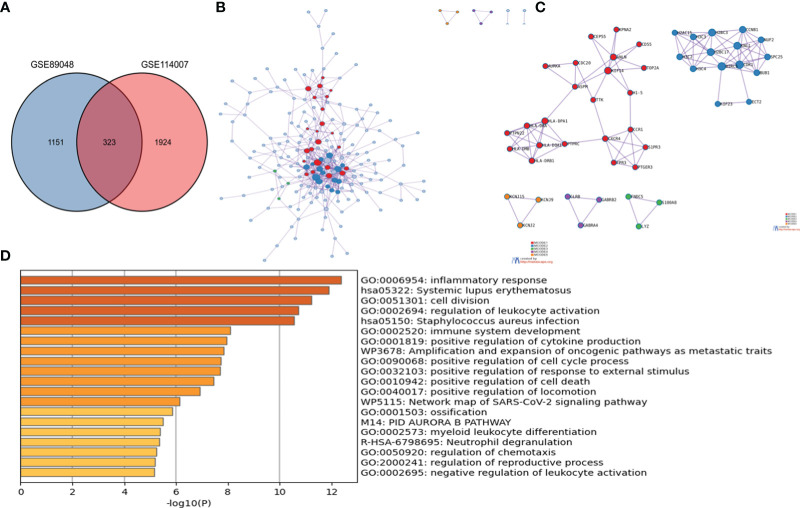
The enrichment analysis of OA-DEG Metascape. **(A)** 323 OA-DEGs was identified by overlapping genes between the DEGs of GSE114007 and the DEGs of GSE89408. **(B)** Protein–protein interaction (PPI) network. **(C)** significant MCODE components form the PPI network. **(D)** Heatmap of Gene Ontology (GO) enriched terms and Kyoto Encyclopedia of Genes and Genomes (KEGG) enriched terms colored by p-values.

323 OA-DEGs was identified by overlapping genes between the DEGs of GSE114007 and the DEGs of GSE89408. By using the Metascape database, the analysis of PPI, MCODE and the function enrichment of OA-DEGs were obtained (**Figure 3**). The results suggested that biological function was primarily related to immune and inflammatory response.

### Screening of immune-related hub DEGs and analysis of functional enrichment

**Figure 4:**
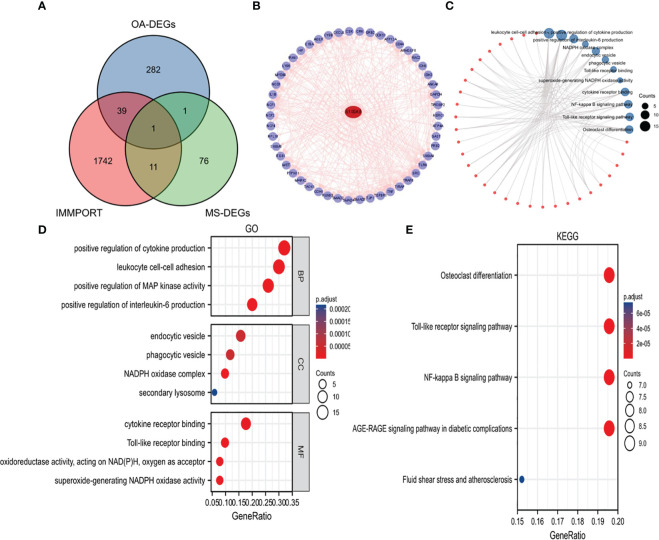
Identification of S100A8 and analysis of 50 targeted binding proteins of S100A8. **(A)** Screening of S100A8 by overlapping genes between IRGs, OA-DEGs and MS-DEGs. **(B)** PPI network of 50 targeted binding proteins of S100A8. **(C)** visual network of GO and KEGG analyses. **(D)** GO analysis. **(E)** KEGG analysis.

We obtained Immune-Related hub DEG S100A8 by overlapping genes between IRGs, OA-DEGs and MetS-DEGs (**Figure 4A**). 50 S100A8 target binding proteins were screened by utilizing the STRING database and Cytoscape (**Figure 4B**). Then, 50 S100A8 target binding proteins were used for GO enrichment analysis (**Figures 4C, D**) and the results revealed that the main biological processes (BP) encompassed positive regulation of cytokine production, leukocyte cell-cell adhesion, positive regulation of MAP kinase activity, positive regulation of interleukin-6 production. The cellular component (CC) was primarily enriched in endocytic vesicle, phagocytic vesicle, NADPH oxidase complex, secondary lysosome. The molecular function (MF) was mainly involved in cytokine receptor binding, Toll-like receptor binding, superoxide-generating NADPH oxidase activity. The KEGG pathway enrichment (**Figure 4E**) was primarily related to Osteoclast differentiation, Toll-like receptor signaling pathway and NF-kappa B signaling pathway.

### Single-gene analysis for the S100A8 in OA database

#### Identification of DEGs and GSEA analysis

**Figure 5:**
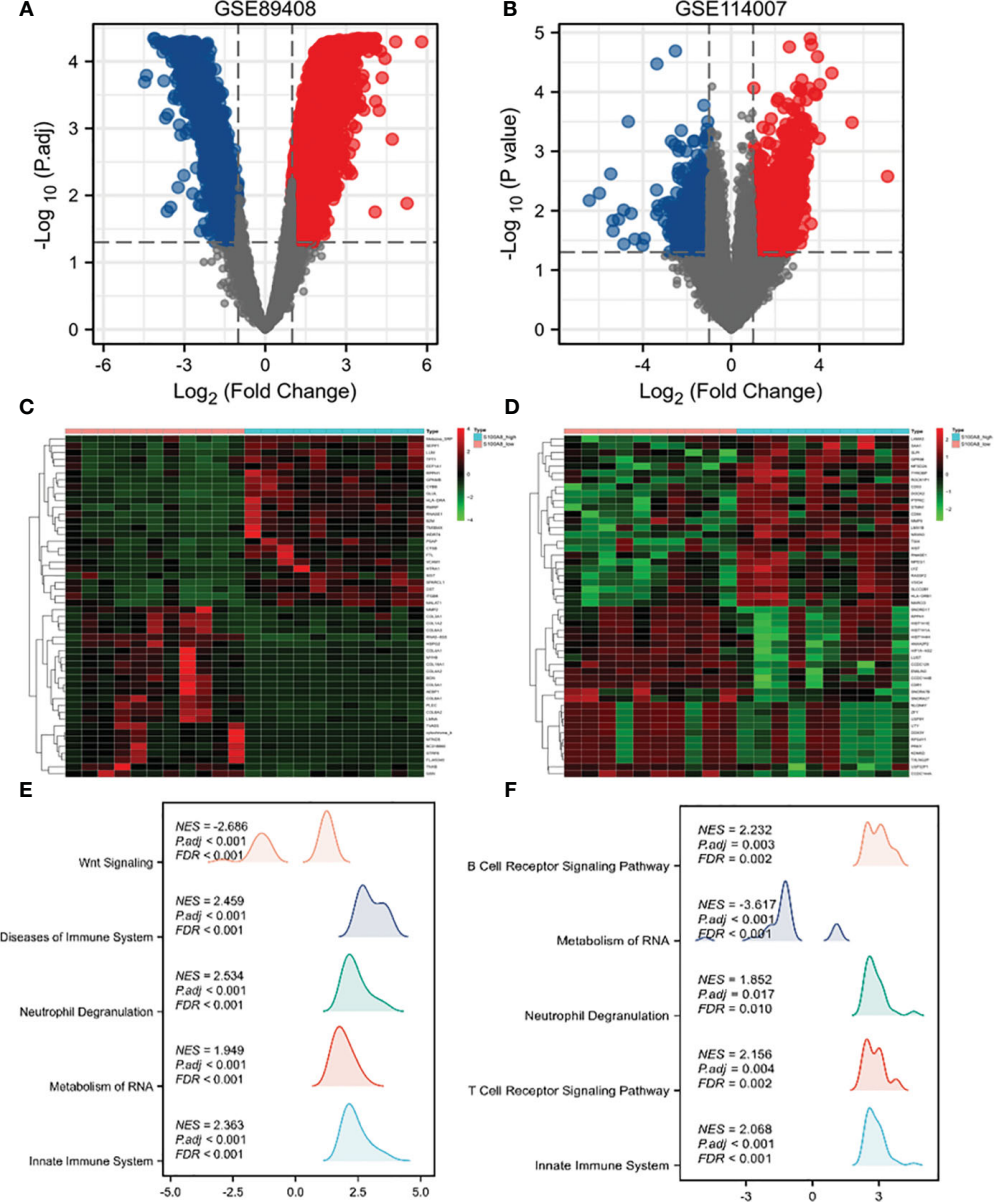
Single-gene analysis and GSEA of S100A8 in OA. Volcano plots of DEGs in S100A8 high-expression group compared to S100A8 low-expression group in GSE89408 **(A)** and GSE114007 **(B)**. Heatmaps showing DEGs in S100A8 high-expression group compared to S100A8 low-expression group in GSE89408 **(C)** and in GSE114007 **(D)**. GSEA between the high- and low-S100A8 expression groups in GSE89408 **(E)** and in GSE114007 **(F)**.

In the OA sample of GSE89408 dataset, there were 7641 DEGs between S100A8 high expression group and S100A8 low expression group, including 5907 upregulated and 1554 downregulated DEGs (**Figures 5A, C**). In the OA sample of GSE114007, there were 2918 genes differentially expressed, including 1958 upregulated and 960 downregulated DEGS. (**Figures 5B, D**).

To identify differentially activated signaling pathways in OA samples of GSE89408 and GSE114007, we further conducted GSEA between the high and low expression of S100A8. In both datasets, enrichment of biological processes associated with immunity More. The most significantly enriched signaling pathways are listed according to the Normalized Enrichment Score (NES) (**Figures 5E, F**). In the GSE89408, GSEA mainly identified reactome innate immune system, reactome metabolism of RNA, reactome neutrophil degranulation, reactome cell cycle checpoints, reactome mitotic metaphase and anaphase (**Figure 5E**). In the GSE114007, GSEA mainly identified reactome signaling by GPCR, NABA matrisome associated, reactome innate immune system, reactome GPCR ligand binding, reactome neutrophil degranulation. (**Figures 5F**).

#### Correlation between S100A8 and immune cell infiltrates

**Figure 6:**
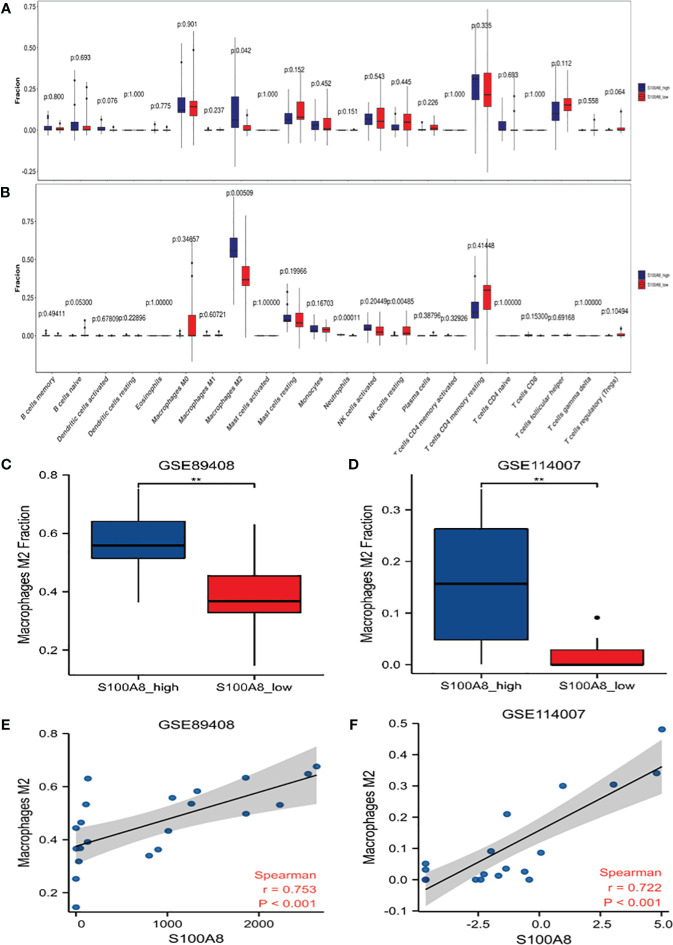
Analysis of S100A8 and Immune Cell Infiltrates. Differences in the levels of immune cells between the S100A8 high- and low-expression groups in GSE89408 **(A)** and GSE114007 **(B)**. Different M2 macrophages infiltration levels among the S100A8 high- and low-expression groups in GSE89408 **(C)** and GSE114007 **(D)**. Correlation analysis between S100A8 expression and M2 macrophage levels in GSE89408 **(E)** and GSE114007 **(F)**. (**p < 0.01).

The immune cell infiltration analysis of OA samples from GSE114007 and GSE89408 datasets both showed that M2 type macrophages infiltration was significantly different between high and low expression of S100A8 groups (**Figures 6A, B**). The enrichment scores of M2 type macrophages in high expression of S100A8 group were significantly higher than those in low expression of S100A8 group in both datasets (all p < 0.01) (**Figures 6C, D**). Moreover, the expression level of S100A8 was positively correlated with M2 macrophages infiltration in both datasets (r=0.753 in GSE89408, r=0.722 in GSE114007, all p < 0.001) (**Figures 6E, F**).

#### Validation of S100A8 expression and diagnostic value

**Figure 7:**
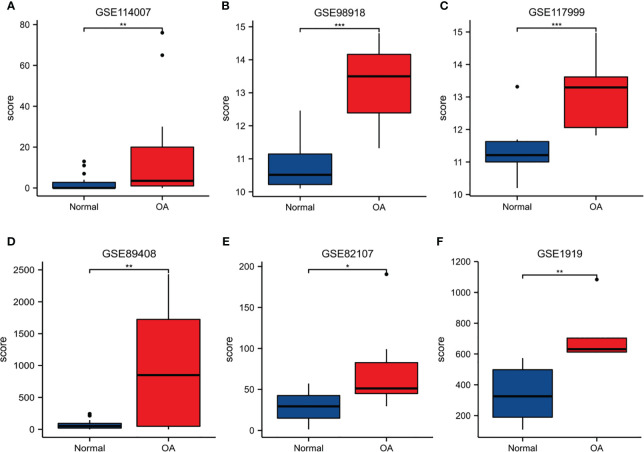
The expression level of S100A8. The S100A8 expression level compared between Normal and OA samples in GSE89408 **(A)**, GSE98918 **(B)**, GSE117999 **(C)**, GSE114007 **(D)**, GSE82107 **(E)** and GSE1919 **(F)**. (*p < 0.05; **p < 0.01; ***p < 0.001).

In order to verify the reliability of S100A8 expression levels, we added other four datasets containing OA and normal samples and analyzed S100A8 expression level. The outcome suggested that S100A8 expression was significantly upregulated in the OA samples compared to normal samples in all six data sets (**Figure 7**).

**Figure 8:**
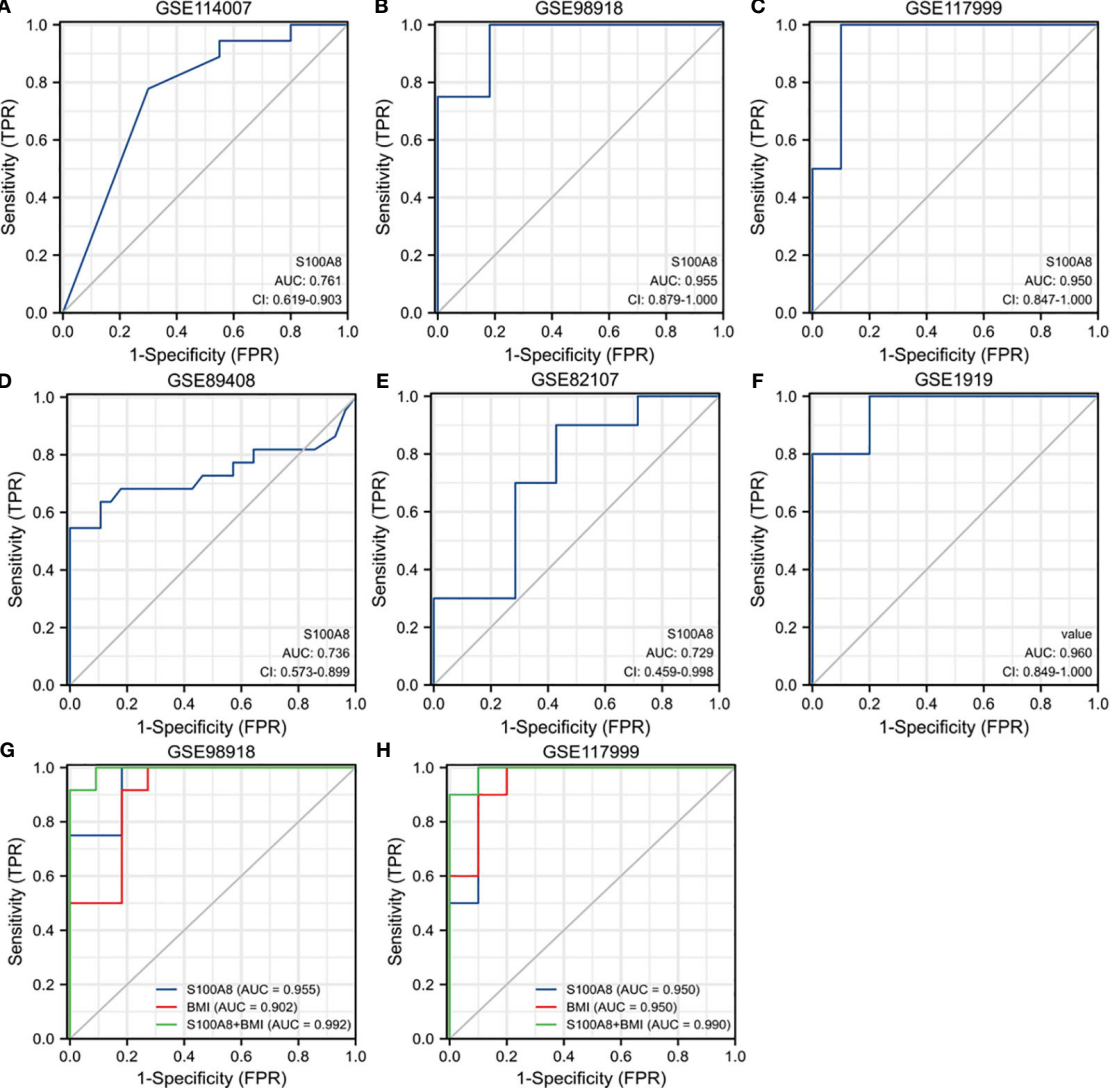
Receiver operating characteristic (ROC) curve for S100A8 expression in OA. The ROC curve of S100A8 in GSE89408 **(A)**, GSE98918 **(B)**, GSE117999 **(C)**, GSE114007 **(D)**, GSE82107 **(E)** and GSE1919 **(F)**. The ROC of S100A8+BMI in GSE98918 **(G)** and GSE117999 **(H)**.

ROC curves were created by using data from the OA samples in the six datasets above versus normal subjects. The results showed that S100A8 was of great value for diagnosis. The AUC of the variable S100A8 were 0.761 (95% CI: 0.619–0.903) in GSE114007, 0.736 (95% CI: 0573–0.899) in GSE89408, 0.955 (95% CI: 0.879–1.0) in GSE98918, 0.950 (95% CI: 0.847–1.0) in GSE 117999, 0.729 (95% CI: 0.459–0.998) in GSE82107, 0.960 (95% CI: 0.849–1.0) in GSE1919. In addition, we found that the combination of S100A8 and BMI has higher diagnostic value for OA. The AUC of S100A8+BMI were 0.992 and 0.990 in GSE98918 and GSE117999 (**Figure 8**).

### Single-gene analysis for the S100A8 in MetS database

#### Diagnostic value and immune infiltration analysis of S100A8

**Figure 9:**
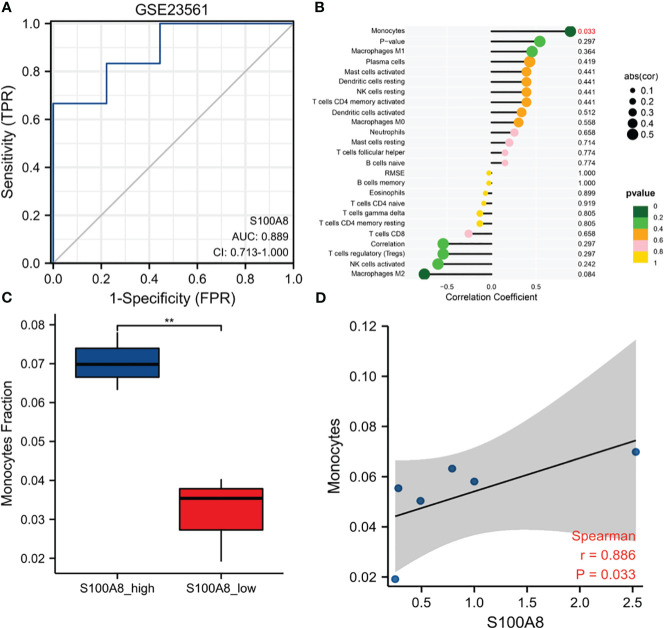
Diagnostic value and Immune Infiltration Analysis of S100A8 in MS. **(A)** The ROC curve of S100A8 in GSE23561. **(B)** Differences in the levels of immune cells between the S100A8 high- and low-expression groups in GSE23561. **(C)** Different monocyte infiltration levels among the S100A8 high- and low-expression groups in GSE23561. **(D)** Correlation analysis between S100A8 expression and monocyte levels in GSE23561. (**p < 0.01).

ROC curves were created by using data from MetS samples and control samples in GSE23561. The outcome suggested that the AUC of S100A8 was 0.889 (95% confidence interval: 0.713–1.0) in GSE23561 (**Figure 9A**). The analysis results of infiltration degree of 22 immune cell showed that the infiltration of monocyte was significantly different between S100A8 high expression group and S100A8 low expression group (**Figure 9B**). High infiltration of monocytes was detected in samples with elevated S100A8 expression (**Figure 9C**). Further validation of the correlation study indicated that the expression level of S100A8 was correlated positively with monocyte infiltration (**Figure 9D**).

#### Validation of S100A8 expression and diagnostic value in metabolic related diseases

**Figure 10:**
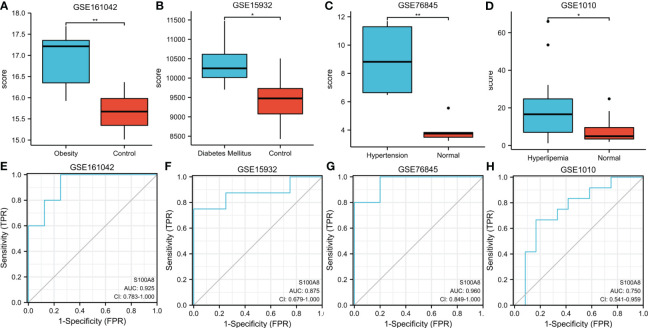
Expression and ROC of S100A8 in Metabolic related diseases. **(A)** The S100A8 expression level compared between obesity samples and Normal in GSE161042. **(B)** The S100A8 expression level compared between Diabetes Mellitus samples and Normal in GSE15932. **(C)** The S100A8 expression level compared between Hypertension samples and Normal in GSE76845. **(D)** The S100A8 expression level compared between Hyperlipidemia samples and Normal in GSE1010. The ROC curve of S100A8 in GSE161042 **(E)**, GSE15932 **(F)**, GSE76845 **(G)** and GSE1010 **(H)**. (*p < 0.05; **p < 0.01).

To further explore the expression and diagnostic value of S100A8 in metabolic related diseases, we verified it in the 4 datasets of metabolic related diseases. The outcome indicated that S100A8 expression levels were significantly higher in four metabolic related diseases, namely obesity, diabetes, hypertension and hyperlipidaemia, than in the control group (**Figures 10A–D**). Moreover, S100A8 also has high diagnostic value for four metabolic related diseases. For obesity, diabetes, hypertension and hyperlipidemia, the AUC values of S100A8 were 0.925 (95% confidence interval: 0.783–1.0), 0.875(95% confidence interval: 0.679–1.0), 0.96 (95% confidence interval: 0.849–1.0), 0.75 (95% confidence interval: 0.541–0.959), respectively (**Figures 10E–H**).

#### Associations between S100A8 expression and body mass index

**Figure 11:**
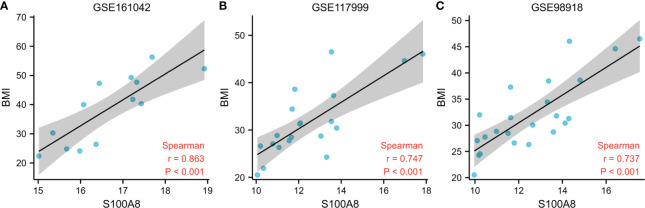
Associations Between S100A8 Expression and BMI. Correlation analysis between S100A8 expression and BMI in GSE161042 **(A)**, GSE117999 **(B)**, GSE98918 **(C)**.

To further explore the association between S100A8 expression and BMI, we analyzed the correlation between the two. The outcome of the correlation study suggested a positive correlation between the expression level of S100A8 and BMI, as shown in **Figure 11**. (r=0.863 in GSE161042, r=0.747 in GSE117999, r=0.737 in GSE98918, all p<0.001)

## Discussion

OA is the most prevalent type of arthritis (14), affecting 1 in 3 older people all over the world (15). MetS is common in the general population and is a major public health problem prevalent worldwide (16). It is a group of metabolic abnormalities that include hypertension, obesity, dyslipidaemia and insulin resistance (16). MetS is also thought to be a comorbid condition of many immune diseases, such as psoriatic arthritis (PsA) and OA (17, 18). No doubt about it, OA and MetS are both leading public health problems with increasing rates of illness and disability.

Notably, MetS provides a higher propensity for the development of OA as a major risk factor for OA (19). In particular, diabetes, obesity, dyslipidaemia and hypertension, as four of the main features of MetS, are precisely the four main metabolic abnormalities that are closely associated with OA (20, 21). So far, plenty of studies have identified inflammation and metabolic abnormalities as two important factors contributing to cartilage degeneration and OA progression (22–24). So it can be inferred that OA may actually be a metabolic syndrome-related disorder with low systemic inflammation originating from metabolic abnormalities. However, the molecular mechanisms between OA and MetS are still not clear. In this study, first of all, to study the relationship between OA and MetS, we used GSEA method to verify the correlation between OA and MetS in GSE89408 and GSE114007 respectively, and found that OA and MetS are positively correlated in both datasets. In order to further explore the possible common mechanisms between OA and MetS, we obtained the common hub gene S100A8 of OA and MetS through differential gene expression analysis. We also found that S100A8 may play an extremely crucial role in the development of OA and MetS.

S100A8 belongs to damage-associated molecular pattern (DAMP) protein family and is mainly produced by activated macrophages, monocytes and neutrophils (25). Early on, researchers found high expression of S100A8/A9 in serum, synovial membranes and synovial fluid of OA patients (26, 27). Another study also showed that serum S100A8/A9 levels were positively correlated with cartilage defects, knee symptoms and increased serum cartilage degrading enzymes in the knee of OA patients, indicating a possible role for S100A8 in osteoarthritis of the knee (28). Furthermore, high levels of S100A8 mRNA were expressed in biopsies from both early symptomatic OA patients and patients with end-stage OA, suggesting prolonged expression of S100A8 throughout the course of OA (28). It has therefore been suggested that S100A8 may be a useful biomarker for predicting and evaluating cartilage destruction in OA, on the one hand because levels of S100A8 are increased in OA and remain high over time, and on the other hand because patients with elevated S100A8 levels are at greater risk of progressing to severe OA (26). For mechanistic exploration, it has been suggested that S100A8 is linked to an increase in cartilage defects and may play an important role in the etiopathogenesis of cartilage defects in OA. We know that the matrix metalloproteinase (MMP) has been shown to trigger cartilage deterioration and facilitate the progression of OA (29), S100A8 may play a role in cartilage defects by upregulating the expression of these matrix metalloproteinases (28). S100A8 induces catabolic phenotype in OA chondrocytes through TLR-4 upregulation of MMP1,3,9,13 and pro-inflammatory cytokines (30). Similar reports have been made in animal experiments. In collagenase-induced osteoarthritic mice (CIOA), S100A8/A9 was up-regulated in synovial membranes and serum compared to control. Moreover, S100A8 mRNA and S100A8/A9 heterodimer stayed at a high level for a long duration (as long as 21 days post injecting) (26). In terms of mechanistic studies, on the one hand, cartilage disruption and synovial activation were decreased in CIOA S100A9 -/- mice (double knockout S100A8 and S100A9 functionally) compared to CIOA wild type mice (26, 31). On the other hand, stimulation of OA donor chondrocytes with S100A8 facilitated articular cartilage breakdown by upregulating (MMP1, 3, 9, 13, IL-6, IL-8 and monocyte chemotaxis protein 1) and downregulating metabolic markers of synthesis (type II collagen and aggrecan) (30).

Similar to previous reports, we also found that S100A8 level was significantly elevated in OA samples compared to controls. We divided the OA samples in GSE114008 and GSE89408 into S100A8 high expression and S100A8 low expression groups and performed GSEA analysis, and the outcome indicated that more biological processes associated with immunity were significantly enriched in both datasets. To learn more about association of S100A8 levels with immune cells, immune infiltration analysis suggested that the enrichment scores of M2 type macrophages in S100A8 high-expression group were markedly increased compared to S100A8 low-expression group. In particular, correlation analysis suggested a significant positive correlation between S100A8 expression levels and M2 macrophage infiltration. We also validated the diagnostic value of S100A8 in six OA datasets and found that S100A8 had significant value for diagnosing.

The role of S100A8 in MetS is not very clear, although chronic inflammation and innate immunity are important factors in the pathogenesis of MetS (32). Of particular note is also the close association of S100A8 with four major metabolism-related diseases, namely diabetes, obesity, hypertension and hyperlipidaemia, and the important role it plays in their pathogenesis. Research has found that a large amount of S100A8 have been found in diabetes patients (33), and it has been used as a biomarker of diabetes (34). Moreover, some studies have found that S100A8 was higher in the obese test population than in the non-obese test population and was reduced by weight loss (35, 36). Circulating levels of S100A8 and visceral adiposity expression are also elevated in obese patients with type 2 diabetes (36). Peripheral blood S100A8 mRNA expression levels significantly associated with visceral fat area, and visceral fat deposits resulted in dysregulated adipocytokine, leading to chronic low-level systemic inflammation (37). So, some researchers have even proposed S100A8 as a novel marker of obesity in non-type 2 diabetics (35). Animal experiments showed that the level of S100A8 mRNA in fat of obese mice was significantly increased. In particular, compared with lean mice, S100A8 expression in white adipose tissue of obese mice was higher (38). In addition, it was also found that in the mouse model, high systemic LDL level would exacerbate OA pathology by causing ectopic bone production and S100A8 generation, the latter leading to increased production of synovial activation and injury-inducible proteins (39).

In the current research, we discovered that S100A8 had high diagnostic value for MetS. Similarly, we divided MetS samples into S100A8 high- and low-expression two groups and analyzed infiltration degree of 22 immune cell. High infiltration of monocytes was identified in the higher S100A8 level samples. Correlation analysis indicated that S100A8 level was correlated positively with monocytes infiltration. Because obesity, diabetes, hypertension and hyperlipidemia were the core of MetS syndrome, we verified the level of expression and diagnostic value of S100A8 in these four metabolic related diseases. The results showed that the expression level of S100A8 was significantly higher in disease group compared to in control group, and all four AUCs suggested that S100A8 had high diagnostic value. Finally, the correlation analysis of S100A8 expression level and BMI level also indicated that they were positively correlated.

Few studies have explored the common hub gene and common molecular mechanism between OA and MetS through advanced bioinformatics approach. Because of the high comorbidity between OA and MetS, we took the lead in exploring and identifying the common hub gene between them, and further conducted single gene analysis, which will help further clarify the relationship and mechanism between OA and MetS. However, our research also has some limitations. First of all, the sample size in the original dataset is somewhat small, and further research should be conducted based on larger samples. Secondly, the function of S100A8 needs to be further verified in clinical models, which will be the focus in our future work.

In conclusion, our results indicated that S100A8 was the common hub gene of OA and MetS, and may participated in the pathogenesis of MetS and OA through immune regulation. In addition, S100A8 was a potential biomarker for diagnosing OA and MetS. Therefore, we supposed that the shared gene S100A8 played a key role in the common pathogenesis of OA and MetS, and may be a therapeutic target in the future.

## Data availability statement

The datasets presented in this study can be found in online repositories. The names of the repository/repositories and accession number(s) can be found in the manuscript/supplementary material.

## Author contributions

Conceptualization: XH. Methodology: XH. Software: XH. Validation: XH and JL. Formal analysis: XH. Investigation: XH. Resources: XH. Data curation: XH and JL. Writing—original draft preparation: XH. Writing—review and editing: XH, JL and WH. Visualization: XH. Supervision: WH. Project administration: WH. All authors have read and agreed to the published version of the manuscript.
